# The role of empathy in shared intentionality: Contributions from Inter-Processual Self theory

**DOI:** 10.3389/fpsyg.2023.1079950

**Published:** 2023-03-10

**Authors:** Elkin O. Luis, Martín Martínez, Kleio Akrivou, Germán Scalzo, Martín Aoiz, José Víctor Orón Semper

**Affiliations:** ^1^Psychological Processes in Education and Health Group, School of Education and Psychology, University of Navarra, Pamplona, Spain; ^2^Methods and Research in Affective and Cognitive Psychology, School of Education and Psychology, University of Navarra, Pamplona, Spain; ^3^Henley Business School, University of Reading, Reading, United Kingdom; ^4^School of Business, Universidad Panamericana, Mexico City, Mexico; ^5^Institute of Modern Languages, School of Education and Psychology, University of Navarra, Pamplona, Spain; ^6^Fundación UpToYou Educación, Zaragoza, Spain; ^7^Francisco de Vitoria University, Madrid, Spain

**Keywords:** empathy, Inter-Processual Self theory, neurosciences, shared intentionality, integrity

## Abstract

Research in psychology related to the conceptualization of empathy has been on the rise in the last decades. However, we argue that there is still space for further research to help capture the important notion of empathy and its theoretical and conceptual depth. Following a critical review of the current state of the research that conceptualizes and measures empathy, we focus on works that highlight the importance of a shared vision and its relevance in psychology and neuroscience. Considering the state of the art of current neuroscientific and psychological approaches to empathy, we argue for the relevance of shared intention and shared vision in empathy-related actions. Upon review of different models that emphasize a shared vision for informing research on empathy, we suggest that a newly developed theory of self, human growth and action–the so-called Inter-Processual Self theory (IPS)–can significantly and novelly inform the theorization on empathy beyond what the literature has stated to date. Then, we show how an understanding of integrity as a relational act that requires empathy is an essential mechanism for current key research on empathy and its related concepts and models. Ultimately, we aim to present IPS as a distinctive proposal to expand upon the conceptualization of empathy.

## 1. Introduction

This study aims to present the reader with a critical review of the literature on empathy and highlights the need for a more holistic vision in the conceptual and research approaches on empathy. Furthermore, it introduces a proposal for a conceptual approach and future research, which is more in line with the interrelational nature of empathy. Therefore, in the present work, the authors wish, throughout the entire document, to promote an open disciplinary dialog on empathy between neurosciences, philosophy and psychology.

To respond to these two objectives, first, the different conceptualization proposals, operating models, measurement instruments, neural substrates, and aspects of developmental psychology will be critically reviewed to summarize the body of literature and draw conclusions on the nature of empathy. In this way, the reader is presented with a comprehensive basis to understand current knowledge on empathy, and the importance of developing new conceptual or theoretical frameworks and new ways of approaching empathy research is highlighted. Then, two fundamental elements in the empathic relationship are introduced: (1) the vision shared between two people and (2) the process of individual but interactive emotional regulation that implies the interaction between two people. Finally, in a subsequent section, the limitations regarding the nature of empathy that previous approaches have identified become evident and a proposal from a new approach is made to investigate and interpret the complexity of empathy: the Inter-Processual Self theory (IPS).

## 2. Literature review

### 2.1. Conceptualizing empathy in psychology

The term empathy has evolved throughout history, and has been conceptualized in different ways, such as (1) knowing the others’ thoughts and feelings (Decety and Jackson, 2004); (2) adopting the posture or expression of an observed other (Bavelas et al., 1986; Preston and De Waal, 2002); (3) imagining how the other feels (Jackson et al., 2006); (4) projecting or imagining what the other thinks or how they feel (Lipps, 1903); (5) imagining how the other is thinking and feeling (Batson et al., 1997); (6) imagining how one would think and feel in the other’s place (Povinelli, 1993); or (7) feeling distress at witnessing another person’s suffering (Krebs, 1975). These conceptualizations have also been referred to as (1) empathic accuracy, (2) motor mimicry, (3) emotional contagion, (4) projective empathy, (5) and (6) perspective or role taking, or (7) empathic distress, among others (see Batson, 2011). Based on their interdisciplinary relevance, the different forms of other-oriented emotion (i.e., feeling for the other) have aroused wide-ranging scientific interest.

Although recent work suggests a meta-definition for empathy, as “the ability to experience the affective and cognitive states of another person, while maintaining a distinct self, in order to understand the other” (Guthridge and Giummarra, 2021, p. 2), a consensus on how to define empathy has not yet been established. Despite the complexity of its definition, empathy’s multidimensional nature is now a given (Baldner and McGinley, 2014), and empathy is expressed in a variety of ways, including as an individual ability, as a personal trait, as a capacity or competence, as a response or reaction to the observation of another’s experiences, and as interpersonal behavior.

In general, empathy represents the psychological response that arises from the need to perceive and understand the emotional state of an interlocutor in order to facilitate care or other-oriented motivation, such as cooperation or socialization (Cheng et al., 2014), although this response can also lead to personal distress. Therefore, it results from the configuration of emotional (sharing affect with the other), cognitive (understanding the other’s subjective state from their point of view), and motivational (feeling concerned for the other) facets (Preston and De Waal, 2002; Decety, 2015).

### 2.2. Theoretical models of empathy

Currently, most of the neuroscientific proposals developed to better understand empathy are framed within Simulation Theory (ST) and Theory of Mind (ToM), which differ in their considerations of empathy. On the one hand, ST claims that empathy is the result of the mental simulation that the observer generates from the interlocutor’s emotional state (Gallese and Goldman, 1998; Gallese, 2003; Rizzolatti and Luppino, 2001). It hypothesizes that “shared representations” of experienced and observed affective, sensory, and motor responses allow perceivers to vicariously experience what it is like to be the target of their perception (i.e., resonate). As an example, the Perception-Action Model (PAM; Preston and De Waal, 2002) considers empathy as the need to perceive what the other is thinking and feeling to subsequently activate mental representations about the interlocutor’s cognitive and affective state (Preston, 2007). Once the subject attends to the state of the object, representations are automatically shared between the actors, and they end up facilitating empathic behavior.

On the other hand, ToM sees empathy as mediated by observer-assigned attributions about the other person’s thoughts and feelings (Premack and Woodruff, 1978). This point of view implies a more complex vision than ST, as it proposes that humans can predict the mental state of others. A related term is empathic accuracy, defined as the extent to which mind-reading attempts are successful (Ickes, 1993). In this respect, Barrett-Lennard (1981) proposed the Cyclical Model of Empathy, which considers the distinct stages involved in the phenomena, and highlights the importance of the interaction processes that form an experiential cycle between the observer and the target persons.

Decety and Jackson (2004) formulated the Multidimensional Model, which aimed to integrate the affective and cognitive aspects of empathy through parallel and distributed processing in a number of dissociable computational mechanisms. This model postulates the dynamic interaction of three components: (1) the interpersonal sharing of affect, (2) self-other awareness, with clear regulatory mechanisms to distinguish between the two parties involved in the interaction, and (3) the cognitive flexibility needed to adopt the other’s perspective. In line with this model, recent work has clarified the importance of affect sharing, emotion understanding, perspective-taking, and emotion regulation in the development of empathy (Decety and Michalska, 2020), emphasizing the key contribution of genetic and environmental factors to the development of empathy and pro-sociality (Knafo et al., 2008).

In experimental terms, the affective priming paradigm is among the most used strategies to investigate how empathy can influence perception prior to subsequent cognitive processing (Zhang et al., 2010). This paradigm is characterized by shorter reaction times between stimuli that are affectively congruent compared to those that are not (Fazio et al., 1986), thus demonstrating how different automatic processes can influence cognitive and emotional processing. In turn, the affective evaluation that the subject makes later on in an event becomes predictable. However, based on methodological constraints, this paradigm still has not been used in experiments because it calls on participants to imagine the empathic state of a subject in relation to the context in which they can be found.

In the Self to Other Model of Empathy, proposed by Bird and Viding (2014), both the perceiver of the situation and the interlocutor supposedly influence the other person’s emotional states. However, the perceiver also has to clearly designate both their experienced affective state, as well as that of their interlocutor. The above model is supported by two systems that facilitate experiencing empathy during interaction. First, the situation-understanding system provides an estimate of the other’s emotional state based on the situation, and is considered an input for the affective cue classification system, which establishes the conditions to produce the corresponding emotional contagion. Furthermore, this model supports the need for the participation of two additional representation systems that are in constant interaction both with each other and with the other two systems: the ToM and the affective representation system. The former represents the states of the self and of others within a propositional system, whereas the latter is responsible for constructing conscious representations regarding the self’s present affective state.

Some authors, e.g., Coll et al. (2017), consider empathy as a state resulting from the functionality of two processes: the identification of one’s own emotions and those of others, and the sharing of affection between the people who interact. In this line, Heyes (2018) presented the dual system model of empathy, which is based on the Learned Matching hypothesis (Heyes and Bird, 2007). In his theoretical proposal, Heyes suggests that people establish connections between situations in which their previous and particular emotional experience from within correlates with their observation of the same emotion in the present situation “from the outside.” In accordance, the empathy process includes two functional systems: automatic and cognitive. The automatic system is based on associative learning, which is developed early in humans and is responsible for (but independent from) the emotional contagion between the people who interact in a given situation. The cognitive system, characterized by being a subject-controlled process, emerges in later evolutionary development and is exclusive to humans. Furthermore, the cognitive system is in charge of working together with the automatic system in one person’s empathic comprehension of another. In short, an emotional stimulus will produce motor and somatic activations that, in turn, elicit an automatic response based on metacognitive and cognitive evaluation resources, which will produce empathic understanding of the interlocutor’s experience. In an attempt to support the development of a coherent theoretical proposal on empathy, an integrative hierarchical model has been recently suggested (Schurz et al., 2021). This model relies on common neurocognitive components engaged by different empathy and ToM tasks, and supports a three-cluster solution, namely cognitive, affective, and intermediate.

In general, most of the above-mentioned models suggest that empathic abilities include cognitive and affective components. The former involves the ability to understand the internal states experienced by another person, whereas the latter refers to an appropriate emotional response to another person’s internal state.

### 2.3. Measuring empathy

Measuring empathy is a serious challenge for researchers in psychology since the concept itself lacks clear definition. As stated by Decety and Jackson (2004, p. 89), “one of the most challenging limits to comparison among studies of empathy stems from the use of different tools and methods.” In general, measurements of empathy can be categorized into three major approaches, including self-report questionnaires (SRQs), behavioral methods, and neuroscientific measurements (Zaki and Ochsner, 2012; Neumann et al., 2015; Bošnjaković and Radionov, 2018).

SRQs consider empathy as a stable state, and represent the common tools used to measure empathy in the general population (see Supplementary Table 1) as they have provided the most comprehensive outcomes to date (Hall and Schwartz, 2019). Widely used SRQs for measuring empathy include Hogan’s Empathy Scale (Hogan, 1969), the Interpersonal Reactivity Index (Davis, 1983), and the Empathy Quotient (Baron-Cohen and Wheelwright, 2004). Other self-report measures of empathy have been developed for target populations, such as children or adolescents (Hashimoto and Shiomi, 2002; Garton and Gringart, 2005; Jolliffe and Farrington, 2006; Dadds et al., 2008), physicians (Hojat et al., 2001), as well as for specific clinical conditions, such as autism (Baron-Cohen et al., 2001), alexithymia (Grynberg et al., 2010), or schizophrenia (Shamay-Tsoory et al., 2007). Although some of the SRQs conceptualize empathy as a multidimensional phenomenon, most of the related questionnaires understand that empathy consists of either one or two components, namely cognitive perception and emotional responsiveness.

Nevertheless, evidence indicates that the factor structure underlying the SRQs that measure empathy responds to multiple subcomponents, rather than to a one- or two-factor solution (Baldner and McGinley, 2014). This evidence is in line with current suggestions on empathy (Hall and Schwartz, 2019), as well as with the meta-analytic conceptualization recently proposed by Guthridge and Giummarra (2021). The self-reported functioning reflects the perception that a person has about his/her performance of tasks, and right or wrong responses do not exist. As a main limit of self-report measures, the results can be overestimation or underestimation of actual ability, so they may not match the actual level of empathy (Coman and Richardson, 2006). Furthermore, SRQs are limited due to response bias, which is produced by subjectivity and susceptibility, and leads to motivational distortion. Indeed, numerous authors have commented on or critiqued SRQ measures of empathy (see Eisenberg and Lennon, 1983; Wispé, 1986; Levenson and Ruef, 1992; Gerdes et al., 2010; Batson, 2011; Baldner and McGinley, 2014; Hall and Schwartz, 2019; Murphy and Lilienfeld, 2019). Their main argument refers to SQRs’ lack of specificity since most self-reporting is narrow in scope; they are also oriented to a specific population and, as a result, may be limited in their capacity to provide valid and reliable interpretations, placing them in a complicated position from a psychometric perspective. One major difficulty that this type of self-assessment tool faces is demonstrating that it reflects a general ability or personality trait, or a temporary condition subject to a greater degree of variation. Consequently, some authors claim that SRQs usually report little about the empathic accuracy of actual behaviors.

In contrast with SRQs, behavioral approaches for the assessment of empathy include evaluations of relevant experimental stimuli and performance on tests, while enabling a focus on interpersonal phenomena (Supplementary Table 2). These approaches have often measured empathy as a reaction to particular stimuli, such as real or imagined people, video clips, or descriptions of situations that might or might not evoke empathy. In such studies, outcomes are coded by an observer who has viewed or listened to a variety of target individuals, or by *in vivo* participants, who rate only one person at a time (e.g., a patient rating their therapist). Following Carl Rogers’ psychotherapy proposal (Rogers, 1957), several authors have suggested that the empathy the therapist shows is a key change process in psychotherapy (Bohart and Greenberg, 1997). Research on the understanding of a therapist’s empathic processes reveals a consensus around the presence of three major subprocesses: emotional simulation, perspective-taking, and emotional regulation. Furthermore, research has also distinguished between three main modes of therapeutic empathy: empathic rapport, communicative attunement, and personal empathy (Elliott et al., 2011). Finally, depending on the rater, the behavioral measures of empathy may fall into four categories: empathy rated by non-participants, client-rated empathy, therapist rating their empathy, and the congruence between the therapist’s and the client’s perceptions of the latter (Elliot et al., 2011).

Examples of widely used behavioral methods for the evaluation of empathy include the Barrett-Lennard Relationship Inventory (Barrett-Lennard, 1962), the Carkhuff Empathy Scale (Carkhuff, 1967), the dyadic interaction paradigm (Ickes et al., 1990), the concern for others scale (Zahn-Waxler et al., 1992), the *faux pas* task (Stone et al., 1998), the emotional attribution task (Blair and Cipolotti, 2000), the affective priming paradigm (Zhang et al., 2010), or the therapist empathy scale (Decker et al., 2014). In addition, different empathy-related interventions have aimed at specific populations, such as physicians (Kelm et al., 2014), pharmacy students (Lor et al., 2015), school-based communities (Lakin and Mahoney, 2006; Castillo et al., 2013), families (Moran and Diamond, 2008; Welton et al., 2008). Finally, recent research has introduced immersive virtual reality to bolster empathy’s understanding (Nascivera et al., 2018; Barbot and Kaufman, 2020). One of the biggest criticisms of behavioral approaches refers to their possible reductionism because they suggest that every behavior can be explained through a stimulus–response relationship, and ignore what cannot be observed, like emotions, internal thoughts, or cognitive biases.

Neuroscientific approaches to empathy include brain imaging techniques like magnetic resonance imaging (MRI), functional MRI (fMRI), or positron emission tomography (PET), as well as other measures of activity in the central nervous system like electroencephalography (EEG), facial electromyography (EMG), or in the autonomic nervous system, like skin conductance and heart rate. Neuroimaging techniques can provide precise information about the spatial localization of the brain structures involved in empathy (Decety and Lamm, 2006). To be relevant to empathic behavior, it is essential that these measures relate brain activity to behavior (Zaki and Ochsner, 2012). Although MRI approaches can measure the neuroanatomical structures that underpin empathy (Banissy et al., 2012), they cannot show the empathic process in action. As an approximation of empathic behavior in action, fMRI and PET studies have used a variety of tasks including feelings and emotions such as pain (Lloyd et al., 2004; Jackson et al., 2005; Zaki et al., 2016), disgust (Wicker et al., 2003; Jabbi et al., 2007; Benuzzi et al., 2008), threat (Nummenmaa et al., 2008), happiness (Hennenlotter et al., 2005; Chakrabarti et al., 2006; Jabbi et al., 2007), distress (Klimecki et al., 2013), emotion valence and arousal (Nummenmaa et al., 2012), as well as cognitive aspects such as mentalizing (Schnell et al., 2011), or perspective-taking (Hildebrandt et al., 2021). In addition, empathy has been induced by means of simple observation (Wicker et al., 2003; Singer et al., 2004; Grosbras and Paus, 2006; Singer, 2006; Krämer et al., 2010; Kanske et al., 2015), imagination (Jackson et al., 2006; Lamm et al., 2007), or the evaluation of the other’s pain (Jackson et al., 2005; Gu and Han, 2007; Moriguchi et al., 2007).

Nowadays, fMRI represents the best method for examining brain function since it is a non-invasive approach that measures deep brain structures with high spatial resolution. However, it has poorer temporal resolution than other measures (such as EEG), and limits subject mobility, which makes it unsuitable for investigating social interactions in natural and ecologically valid setups. EEG has been employed to investigate empathy for pain (Fan and Han, 2008), empathic concern, and positive empathy (Light et al., 2009; Morelli et al., 2015). EEG measures have relatively poor spatial resolution but excellent temporal resolution, matching time-locked stimulus presentations with neural activity. In contrast, physical movement and eye blinks can interfere with EEG recordings. Facial EMG measures provide a non-verbal index of motor mimicry that underlies empathic response, and have demonstrated concurrent validity with SRQs on empathy (Harrigan et al., 2008). Nevertheless, it is important to ensure that any motor mimicry recorded through facial EMG reflects empathic stimuli rather than other kinds of stimuli (Larsen et al., 2003). Finally, heart rate and skin conductance responses have also been proposed as good indicators of autonomic activity associated with empathy (Krebs, 1975; Eisenberg et al., 1994; Zahn-Waxler et al., 1995).

In contrast with traditional paradigms that understand cognition as passive information processing, novel neuroscientific approaches, called “second-person neuroscience,’ understand the mind as an embodied dynamic system (Gallotti and Frith, 2013; Schilbach et al., 2013). In such approaches, perception represents an active process executed by an organism located in the environment, wherein subjects are not isolated from, but embedded within, the perceived world (Thompson, 2010). A central claim in such approaches is that, when we are emotionally engaged and interacting with someone, social cognition differs from when we are detached and merely observing. Consequently, emotional outcomes that rely on proprioceptive afferents from the body and action-based processes are likely to be closely linked and interact in complex ways. Said paradigms demand that emotion be taken as central to mind awareness, and they focus on affective responses rather than reflections or constructs.

Recent studies employing hyperscanning techniques have characterized brain responses during real-time social interaction between two or more people (Kingsbury and Hong, 2020). For example, Schippers et al. (2010) investigated the degree to which two brains resonate during gestural communication *via* the use of hyperscanning fMRI, while demonstrating evidence for resonance theories (Gallese and Goldman, 1998; Rizzolatti and Luppino, 2001). Similarly, Anders et al., 2020 used pseudo-hyperscanning fMRI to investigate the flow of affective information between the brains of senders and perceivers engaged in ongoing facial communication of affect. They found that the perceiver’s level of neural activity can be predicted from the neural activity in the sender’s brain depending on the emotion that is being communicated at that moment, thus supporting current theories of intersubjectivity (Gallese, 2003). In line with this, Kinoshita et al. (2019) conducted an experiment in which participants communicated using facial expressions of joy, sadness, and neutrality while EEG signals were simultaneously recorded. Their results demonstrated the possibility of measuring affective sharing in response to emotional faces, based on specific brain correlation patterns.

In addition, empathy measurement has enabled the identification of distinct genetic, neurobiological, and behavioral moderator variables, such as attention level to stimuli (Gu and Han, 2007), prior knowledge (Lamm et al., 2007), group membership (Xu et al., 2009; Hein et al., 2010; Weisz and Zaki, 2018), affective attachment (Singer et al., 2004), emotional state (Nummenmaa et al., 2012; Silani et al., 2013; Kanske et al., 2015); gender (Eisenberg et al., 1994; Baron-Cohen and Wheelwright, 2004; Jolliffe and Farrington, 2006), intelligence, personality, and socioeconomic status (Jolliffe and Farrington, 2006; Hittner and Haase, 2021), or the propensity to physiological hyperarousal, negative thinking, or chronic exposure to negative parental affect (Tone and Tully, 2014). Crucially, empathy exhibits indirect but strong contextual dependence, shifting between automaticity and context dependency, as a function of how empathizers and situations are characterized (Zaki, 2014; Weisz and Zaki, 2018; Cameron et al., 2019). A key feature of empathic context is its motivation to drive people to avoid or to approximate engagement with the others’ emotions. Empathic motives, such as positive affect, affiliation, and social desirability, encourage people to approach empathy, whereas avoidance motives, such as suffering, material costs, and interference with competition lead people to avoid empathy.

### 2.4. Neural substrates and cognitive networks of empathy

Several EEG studies have implemented paradigms aimed at elucidating the neural mechanisms of empathy by categorizing either the facial expressions (Werheid et al., 2005; Choi and Watanuki, 2014), the exposure to the other’s pain (Jackson et al., 2005; Meng et al., 2012), the emotions of others during cooperative or non-cooperative interactions (Balconi and Vanutelli, 2017), or the perception of negative emotions (Ito et al., 1998). Those studies provide evidence of a negativity bias in affective processing, which reflects a tendency for negative events to generate greater mobilization of physiological, emotional, and cognitive resources in individuals (Cacioppo et al., 1997; Kahneman and Tversky, 2013). However, as far as we know, there is insufficient evidence for the implementation of paradigms involving states of empathy facilitated by positive emotional aspects. Studies combining several of the previous strategies have also been performed (Cao et al., 2015). Nevertheless, they have not achieved the objectives intended herein.

On the other hand, other studies have emphasized evaluations of situational contexts in addition to the search for the neural centers associated with empathy. Some paradigms have been developed that include attribution judgment as a mediator of a person’s empathy responses. Sessa et al. (2014) evaluated Event-Related Potentials (ERPs) in a paradigm that linked facial reactions to white faces (the participant’s own race) and black faces (another race) when faced with a painful condition. The findings allowed them to neurally and temporally define two stages of processing that underlie empathy: a stage biased by race (280–340 ms), when the neuronal responses to pain of individuals of one’s own race vs. another race are amplified; and another stage of cognitive assessment of pain (400–750 ms), without any racial prejudice present. Similar results were obtained by Gutsell and Inzlicht (2012) and Cao et al. (2015) with fMRI.

These studies show that empathy necessarily implies an exchange of emotional, motivational, and cognitive states that not only become manifest, but also regulate one another. This finding leads us to consider what happens when a subject has to think about the conditions in which another person lives, a process of context evaluation that can easily be the first step for later, helpful behaviors.

In this regard, recent studies have indicated that the breadth of late positive potentials (LPPs) are sensitive to emotional reassessment (Hajcak and Nieuwenhuis, 2006). Emotional reassessment implies generating alternative interpretations of emotional stimuli to make them less negative, which may be related to the integration of information prior to coming to an empathic decision. However, it remains unclear whether reductions in negativity related to reassessment in LPPs reflect a decrease in emotional processing or an increase in cognitive demands after reassessment. Foti and Hajcak (2008) used an emotional paradigm to examine the modulation of the electro-cortical response produced by visualization of history prior to the presentation of emotional images (situations or people in neutral or unpleasant conditions). These images were followed by stories that emphasized the simple description of a scene or person, or highlighted the negative aspects of the scene or person. Their findings provided evidence to support the hypothesis that stimulus descriptions are capable of influencing brain activity patterns.

Yet, although it is relatively clear in the literature (1) that people with a higher degree of empathy pay more attention to emotional details that facilitate detection of fundamental facial expressions to understand others’ emotional state and intentionality (Jabbi et al., 2007; Choi and Watanuki, 2014), (2) that expression discrimination modulates LPPs (Weinberg et al., 2012), and (3) that reassessment can be a strategy implemented in situations of uncertainty or of higher affective-cognitive costs associated with empathic decision-making (Foti and Hajcak, 2008), several questions remain unresolved. One of them refers to what happens when an observer has to imagine what their interlocutor thinks or feels about the living conditions imposed on them; that is to say, given the conditions in which they are forced to experience life, what happens when someone has to assess how empathetic another person may be? Empathy is much more than distinguishing an emotional state as it implies a more complex approach, where recognizing and distinguishing emotions are integrated within the speculations that it may trigger about the interlocutor’s emotions and thoughts. This invites us to design and implement a paradigm that takes the subject out of the initial empathic relationship (“I feel empathy for the other in the framework of a relationship of mutual exchange”) and puts that subject in a situation where they are the one who values the empathic relationship that occurs between them and the context (context evaluation state).

Faithful to the dual system model of empathy (Heyes, 2018), some authors, e.g., De Waal (2008), suggest that the basic system of emotional contagion is associated with the lower frontal cortex (Seitz et al., 2008) and the right insula (Wicker et al., 2003). These regions are supposed to be responsible for the executive functions that enable the recognition of the other’s emotional state and the generation in the observer of concern for their interlocutor’s emotional empathy (Dapretto et al., 2006; Schulte-Rüther et al., 2007). Likewise, other studies regarding pain empathy have shown activations in the anterior cingulate cortex, basal ganglia, cerebellum, and brainstem (Singer et al., 2004; Decety and Michalska, 2020). The activation of the anterior cingulate cortex, which is communicated with the anterior insula, has been associated with the processing of mental states between interlocutors (Frith and Frith, 2006; Vaughn et al., 2018). In addition to these areas, it has been observed that the activation of the premotor cortex, inferior parietal lobe, and superior temporal sulcus are relevant to the functioning of the automatic response system and to the emotional contagion within empathy (Zaki and Ochsner, 2012; Heyes, 2018). Furthermore, a more cognitive system has been related to the ventromedial prefrontal cortex (Mitchell et al., 2006) and to the temporoparietal junction (Samson et al., 2004; Strombach et al., 2015), whose function is to mentalize the emotional situation that conditions the other interlocutor and to understand the latter’s perspective (i.e., the understanding of emotions that relies on self- and on other-awareness). Recent proposals have further suggested that empathy can be deconstructed into a process model that includes bottom-up processing of affect sharing, and top-down processing in which the perceiver’s motivation, intentions, and self-regulation influence the extent of an empathic experience. Therefore, the basic macro components of empathy considered in these recently proposed models include emotional contagion, emotion recognition, perspective-taking, caring for others, and emotion regulation (Decety and Michalska, 2020).

### 2.5. The development of empathy

Among the factors that play a role in the development of empathy we find within-child contributions, such as genetics (*via* de neuropeptide oxytocin, see Panksepp, 2004) in concert with environmental factors (Zahn-Waxler et al., 1992; Knafo et al., 2008), factors associated with neural development (Preston and De Waal, 2002), and temperament; socialization factors (imitation, parenting, or parent–child factors). Other social outcomes associated with the development of empathy are the internalization of rules, prosocial and altruistic behavior (Decety et al., 2016), social competence (Cikara and Van Bavel, 2014; Allemand et al., 2015), and relationship quality (McDonald and Messinger, 2011).

The development of human empathy across the lifespan is characterized by (1) an automatic bias to share affect and emotions with others, and then by (2) the development of cognitive processes of perspective-taking and executive control, which enable people to intentionally adopt a subjective perspective of another without confounding the self and the other.

When young children have to confront the distress of another person, they are capable of showing distinct sophisticated behaviors related to empathy, despite their limited verbal expressiveness. Several studies have shown that contrarily to a variety of control stimuli, newborns exposed to the sounds of other’s infant crying display stronger distress reactions, an effect which has been referred to as reflexive crying (Simner, 1971), empathic distress (Sagi and Hoffman, 1976) or distress crying (Martin and Clark, 1982). The specificity of reflexive crying to the sounds of other infants’ wailing supports the hypothesis that there is a biological propensity for interest in and receptivity to the negative emotions of others, which suggests that such feelings of distress responding to others’ negative emotional experiences during infancy may be the precursors to empathic concern (Hoffman, 1975).

Young infants tend to engage in behaviors such as self-comforting in an effort to face being overwhelmed by the negative emotions of others, and thus trying to reduce their personal distress. The development of empathy in children is characterized by the evolution of self-other differentiation, perspective-taking, and emotion regulation during the second year of life, together with a transformation from concern for the self to being capable of concern for the other (Knafo et al., 2008). Thus, empathy-related behaviors such as empathic concern, hypothesis testing, and prosocial behavior are thought to be developed over the second year of life (Zahn-Waxler et al., 1992; Knafo et al., 2008). By 18 to 20 months of age, children are capable of a wide variety of helping behaviors, such as verbal comfort and advice, sharing, and distracting the person in distress; whereas by the third year of life, children are capable of expressing verbal and facial concern and interest in the other person’s distress.

As children get into the preschool and elementary school years, significant gains are thought to be produced in cognitive empathy, particularly due to empathic reflection facilitated by language abilities. By preschool age, children are capable of taking another’s perspective in false belief tasks. The ability to understand others’ perspectives is integral to fully and successfully identify themselves with another’s experience, and allows children to engage in more effective helping strategies, as they presumably view the situation more accurately.

Therefore, it has been proposed that both aspects of empathy typically occur together during the development of a child, and that unequal developments may lead to social dysfunction. Eisenberg et al. (1999) examined longitudinally the stability and consistency of prosocial responses in children from 4 to 20 years of age. They reported that observed spontaneous sharing predicts prosocial disposition, whereas empathy-related responding partially mediates this relation (Eisenberg et al., 1999). Knafo et al. (2008) suggest stable individual differences in empathy-related behaviors during early childhood.

## 3. The importance of a shared vision in empathy

An empathic relationship inevitably implies a quality relationship, that is, a relationship where two persons consider mutual help as part of a common vision (i.e., shared vision). In a special issue paper, Boyatzis et al. (2015) suggest as indicators of a quality interpersonal relationship that its members are compassionate to one another, share a positive state of mind, and especially, that both participate in a shared vision within the relationship. The authors consider this last variable the strongest indicator to define a quality relationship, since a shared vision involves and amplifies the Positive Emotional Attractor (PEA). This attractor is understood as a state composed of physiological, neurological, psychological and emotional characteristics, which facilitate a positive cognitive, emotional, and behavioral state in people. This positive state, in turn, enables openness to the development of new ideas between the people involved in the focal relationship. The PEA has its negative counterpart in the Negative Emotional Attractor (NEA), which predisposes those involved toward experiencing a negative cognitive, emotional, and behavioral state.

Boyatzis (2009) model of attractors, the PEA is characterized at a physiological level by greater parasympathetic influence, the release of vasopressin and oxytocin, linked to social bonding, decreased blood pressure, and by increased heart rate variability. The NEA, for its part, is characterized by greater sympathetic influence, the release of norepinephrine, epinephrine, and cortisol, which are neurotransmitters associated with defensive behaviors, and by an increase in both blood pressure and respiratory rate. At the neurological level, the PEA is supported by the activation of the default mode network (DMN) contrary to the NEA, which is supported by the Task Positive Network (TPN). On an emotional level, the PEA is defined by positive affect (hope, joy, fun, or euphoria), whereas the NEA depends on negative affect (defensiveness, guilt, shame, fear, or anxiety).

At the cognitive level, the PEA improves the performance of working memory and the person’s perceptive openness, as well as it directs the individual to a regulatory state, which drives the individual toward a future state of promotion. Said state of promotion consists of a personal vision centered on a so-called ideal self, which motivates an individual to address situations that are consistent with their personal vision, that is, with their personal growth and ideals, and that are in harmony with their values (Higgins, 1997). On the other hand, executive functioning is reduced in the NEA, and the person is guided to a more preventive regulatory state. In this regulatory state, one’s personal vision is more focused on what should be, on security needs and no-loss situations (Higgins, 1997). For some authors, e.g., Boyatzis et al. (2015), the effective vision is more focused on the so-called “ideal self” (promotion) instead of on the so-called “ought self,” the one in which a person should be consistent with others’ expectations (prevention), which functionally leads to desired and sustained change over time.

Finally, at the relational level, the PEA allows for an orientation toward learning and greater receptivity vis-à-vis one’s interlocutor, while the NEA presents the opposite situation, whereby the person is more oriented toward their performance and is distant from others. In short, a balance between both “attractors” (PEA and NEA) is necessary for the construction of a shared vision that leads to desired and sustained personal or interpersonal change. However, reality reveals that people may be spending much more time in the PEA than in the NEA (Boyatzis et al., 2015). Although the NEA is necessary to mobilize a person to action and is more appropriate for routine tasks, rather than ones involving more adaptation and learning (Seijts et al., 2004), staying within that attractor can also be counterproductive for mental health and performance, as individuals stuck there predominantly experience negative emotions (VandeWalle et al., 1999).

A vision becomes shared when people spread it to their interlocutors and vice versa, which creates a space where sharing predominates for the sake of developing a common perspective for a wider group. Understanding the appropriate levels associated with PEAs/NEAs makes it possible to identify people’s optimal adaptative conditions, allowing them to open up (to their interlocutor) and, therefore, to share their ideas for creating a shared vision. This implies that in a state of shared vision and when providing effective help, the interacting persons must be open to informing and listening to their own needs, concerns, or desires, as well as to those of their interlocutor(s). The attractors help to show why focusing on problems can awaken a NEA state by which the person closes in on themselves. In such a state, the person might have thoughts like, “I do not think I will be able to help my friend,” “Another person will be more effective in helping you,” or “I cannot help this person because I know I am not going to do it right” and, therefore, the empathic behavior will not occur, or will not occur in a timely fashion. Now, the person operating according to a PEA, may be open to searching for help-oriented, prosocial action alternatives and have thoughts like “We can do things to solve it” or “I do not know what to do now, but I do know who can help us, and I’m going to call them.”

However, empathic processes are best situated in light of balanced, personal activation between the two attractors (PEA/NEA). The PEA allows one to adapt to complex situations where help is sought, and enables openness to find appropriate action options, whereas the NEA guides the person to act accordingly, and forces them not to seek help and to close oneself off from possible solutions. But the joint work between the two attractors (personality, temperament, and character variables) can enable both openness to the exploration of creative options and evaluation of the helping behavior. This includes how to avoid the potential risks involved when there is a willingness to help but not the necessary skills to do so, that is, it likely facilitates more prudent and effective helping behaviors (Gibson and Sanbonmatsu, 2004).

## 4. Empathy enters emotional regulation and integrative proposals

Traditionally, emotional regulation has been defined as the process by which people steer their own emotions, either in the moments in which they have those feelings, or in the way in which they experience and express them (Gross, 1998, 2015). Emotional regulation is essential for empathy since it facilitates the distinction between one’s own emotions and those of the other person (Filippetti et al., 2012). When people empathize, they need to regulate the emotions evoked, so that an affective overflow that could impact possible prosocial, helpful, or cooperative behavior does not interfere.

Although the association between emotional regulation and the empathic process is widely accepted (Thompson et al., 2019), research on this relationship has focused almost exclusively on an intrapersonal perspective. In fact, since the end of the twentieth century, different models have been developed that focus on people’s ability to regulate their emotional states (Reeck et al., 2016). For example, the regulation process model (Gross, 2015) considers the existence of four components in the process of generating emotions: (1) a relevant situation from which (2) attentional processes and (3) thoughts directed at the specific situation emerge and finally, (4) an emotional response. With these four components, the person can understand their emotions and organize regulation of the emotional processes involved in each of the components, i.e., the situational component. They can also try to modify the situation. For attention, this involves diverting attentional focus toward other lived aspects of self-experiencing; for thoughts, they can try to reassess the situation, and, for emotional response, they can adapt how the emotions felt are expressed (Gross et al., 2000). However, these types of models do not consider the others involved in the interpersonal relationship or the social characteristics of regulation. It seems that abundant research with an intrapersonal perspective on regulation has led to a lack of research on interpersonal regulation (Niven et al., 2009; Van Kleef et al., 2010).

Recent studies on emotional regulation have emphasized the role of social processes within emotional regulatory mechanisms (Williams, 2007; Van Kleef et al., 2010; Zaki and Williams, 2013; Niven et al., 2019a). In this sense, interpersonal relationships (IRs) constitute a fundamental research paradigm to recognize the implications of co-regulation between people or identify shared patterns of affective oscillation between individuals. According to Gross (2015), these new approaches promise to highlight the role of emotional regulation in social relations and vice versa. For this reason, interpersonal emotional regulation is understood, in the context of an interpersonal relationship, as the process by which interlocutors influence each other. Maintaining or modifying, first the occurrence itself, and then the intensity and the duration of each one’s emotional states (Niven et al., 2019b), and impacting their lived relationship. This co-management of emotional responses contributes to the maintenance of mental health and the improvement of IRs. For Niven (2017), when two actors that are speaking empathically (when two actors are within an empathic dialog) they are linked in the interpersonal emotional regulation process: the target, who has not achieved emotional balance, and the regulator, who assists the target in their emotional regulation process. In this interaction, four characteristics of interpersonal regulation are evident: (1) the process is goal-oriented (to the other person); (2) its purpose is affective, since it influences the feelings of the interlocutor; (3) it is rather conscious, controlled, and deliberate; and (4) it fulfills social functions.

In line with the above-mentioned research, some authors, e.g., Thompson et al. (2019), believe that there is sufficient evidence to consider empathy and emotional co-regulation as intrinsically related. Different studies have identified the overlapping of neural areas associated with reappraisal as an emotional regulation strategy, with regions involved in the cognitive component of empathy (Sabbagh et al., 2006; Urry, 2009). Likewise, the ability to understand the interlocutors’ emotional states, as measured by self-reports, positively correlates with the frequent implementation of reappraisal (Powell, 2018; Thompson et al., 2019). However, although the importance of interpersonal regulation is recognized, few studies have tried to integrate aspects related to intrinsic regulation (i.e., the phenomenon whereby a person initiates social contact to regulate their own emotional experience), or extrinsic regulation (i.e., the phenomenon whereby a person tries to regulate the emotional experience of another) into a conceptual model of interpersonal regulation to explain its potential effects on the dynamics of emotional experiences, such as empathic processes, altruistic behaviors, or cooperation.

In this regard, some authors, e.g., Zaki and Williams (2013), point to the fact that the (intrinsic or extrinsic) mechanisms related to interpersonal regulation interact with two other processes which are either: (1) response-dependent, or (2) response-independent. Response-dependent processes occur when, for example, a person feels better after expressing their emotional state to another, but only if that other person is supportive. Alternatively, independent response processes, where the interlocutor need not respond in a specific way, appear when, for example, a person identifies their emotions and facilitates their regulation during interaction with another person, that is, when the regulation is independent of the response. These authors believe that people participating in an interaction simultaneously implement intrinsic and extrinsic regulation strategies by manifesting behaviors that are either dependent or independent of their interlocutors’ response(s). Therefore, IRs involve the simultaneous deployment of intra- and inter-personal regulation processes. For example, people usually modulate their expressive behavior (facial gestures) when they are in the presence of others (Jakobs et al., 2001) and, similarly, they often share their emotional states with those that they believe can help them (Collins and Feeney, 2000). These findings may suggest that empathic IRs reflect intrinsic and extrinsic regulatory objectives among the people involved and, likewise, that IRs involve dependent or independent response mechanisms within the framework of the interpersonal relationship.

## 5. Limitations of current research on empathy

The first limitation of the research on empathy corresponds to the fact that most related experimental paradigms present confusion between emotion identification and affect sharing, which have often been used as interchangeable terms (Coll et al., 2017). Thus, future research should attempt to properly characterize each of these mechanisms and their interactions to improve our understanding of the empathic process.

Secondly, methodological and technical limitations associated with the research on empathy have forced researchers to start from conceptual definitions and operationally define them in order to best approximate them under experimental conditions. Thus, researchers are faced with having to segment the construct into measurable components, which, in turn, makes it possible to draw specific inferences about each sub-process, but at the cost of oversimplifying the whole empathic process. The first approaches to evaluating empathy in psychology and the neurosciences started from quantitative measurements of a subject in relation to a task, which fails to consider the interactivity of face-to-face IRs. An empathic response is the result of a dynamic process that evolves over time; it presents the possibility of moving from later stages of processing and feedback to their earlier versions and highlights the crucial role that the interpersonal-relational interaction plays in empathy. As past quantitative experiments have failed to address this aspect, this omission remains glaringly obvious when viewed from a humanistic perspective on relationships.

However, more recently, the most interactive aspects of IRs framed in empathy have been addressed. Studies such as those carried out by Huang et al. (2016) or Kinoshita et al. (2019) evaluate the recognition of emotions and their impact on the sharing of affection between two people simultaneously monitored by a hyper scanning EEG. Furthermore, these studies have explored the possibility of creating situations that might generate possible misinterpretations of the actions between the participants and their possible effects on the realization (or not) of empathic behavior. These conditions can lead to the identification of potential meta-cognitive conditioning of empathic processes that facilitate or hinder IRs, such as wanting to control the other, wanting to control the situation, obtaining reinforcement for the behavior emitted, avoiding punishment for such behavior, invading the intimacy of the other in order to help, or forbidding or refusing help from the other with the intention of maintaining autonomy. The last examples represent distinct circumstances that are only observed in the interaction between two people, and that have not been studied because of the technical difficulties involved in carrying out laboratory-based studies on IRs.

## 6. Integrity, empathy and the Inter-Processual Self theory

IPS theory proposal is a cross-disciplinary conceptualization of self and action (Akrivou and Orón, 2016; Akrivou et al., 2018) which draws mainly from the works on transcendental anthropology of Polo (1971) and Polo (1999) and that is rooted in moral psychology. In accordance, IPS postulates four general propositions which allow us to understand its conceptualization of empathy.

*Each person is a singular and transcendental ‘whole’ being. As such integrated complicated totality, a person represents more than the addition of her parts, actions, or states.*

IPS is based on human development theory and the unity between knowledge and action (Frisina, 2002). According to IPS, each human being can be understood in an in-depth way only as a transcendental being characterized by a profoundly complicated singularity: one is more than the addition of their parts and more than their biological nature and their selfhood and identity (Akrivou et al., 2018; Luis et al., 2021). Indeed, looking at its philosophical roots (Polo, 2016), a person is a transcendental, coexistent being (Polo, 2015); a complicated singularity who it is *more than* (i.e., “besides”) her actions. As such, a person *is* already integrated (as opposed to having integrity), from within this personal integrity emerges personal action. Hence a person is considered as a unified system, which forces her to reject any type of artificial division of her constituent parts or processes and implies a more holistic vision of said processes should be accepted.

*Since a person is a singular, complicated already integrated ‘whole’ and a co-being, further human growth requires personal effort for a growing integrity in/through IRs premised upon each person’s action and growth in intimacy.*

Hence, integrity in IPS is premised on human and personal development, whereas growth happens through the person in their integrity which is relational in nature rather than an autonomous project— since the self is understood neither as an autonomous nor as an autonomous-processual subject-agent— but rather as a co-being person (Polo, 2016) which requires personal effort for growing integrity in/through IRs premised upon each person’s mature action (Akrivou and Orón, 2016). This is so because for IPS the person grows through increasing personal relational intimacy with other persons, each in their own singularity and under a conceptualization of personal freedom as “freedom for” which poses each person-actor with the challenge to resolve: how to give effective responses to human co-existence (Polo, 1999). Accordingly, in IPS this personal growth process has to manifest in IRs as a genuine ongoing, and very personal questions involving how to relate and live in integrity while enabling that growth in all related parsons occurs.

*Understanding personal freedom as ‘freedom for,’ free choices emanate for each person in order to effectively respond to human co-existence facing other concrete persons in their reality. Accordingly, humans are guided by the desire and aspiration to integrate three different roots of being: (1) human person, free system premised upon intimacy; (2) virtuous being striving to perfect oneself via growth in virtue with others in the community; and (3) subject-agent producer of novelty under the personal root of being, and concerned about affective intimacy in IRs which enable virtuous growth in a context of shared intentionality.*

To achieve such *integrity* each human being engages in *personal action*. According to IPS theory, personal action with integrity characterizes the self and growth through the following conditions, which constitute desires and aspirations:

Firstly, the person in her choices aims to effectively integrate three fundamental roots or radicals which in the history of philosophy explain human action, namely: (1) the Greek or classical radical— or “radical of nature” in philosophy terms— which is concerned with how each individual as a social being can improve further from one’s biological and cultural origins and how to perfect oneself by virtuous growth both as end in itself and as purpose to contribute to others and the common good; (2) the modern radical— “radical of the subject” in philosophical terms— concerned with achieving novelty through production, and cognitive mastery; and, (3) the “radical of the person” rooted in Christian philosophy tradition, which emphasizes the human person as a free transcendental being characterized by singularity and uniqueness and whose constitution is relational intimacy (Polo, 1999). IPS posits that through the integration of these three roots of being we can better capture all that it means to live and grow as a human being within relations of integrity which connects both the person and others (Polo, 1971; Polo, 2016).

Secondly, in IPS the person in her action aiming to achieve integration of these three radicals can “grow well” only insofar as she can “act well” from within the radical of the person (Akrivou and Orón, 2016) while integrating the “classical” and the “modern” radical. Drawing from Polo’s transcendental anthropology IPS theory recognizes the superiority of the “radical of the person,” which orients and provides meaning to personal action as gift-love while freeing new possibilities for personal and interpersonal growth.

Thirdly, integrity in IPS is concerned with affective intimacy in virtuous IRs involving cognitive, emotional, and ethical-moral processes which enable mutual growth in a context of shared intentionality (Akrivou et al., 2020b). As ethical growth occurs in IRs while it is a quality of an entire person’s personhood, and at the same time optimal cognitive and emotional processing is important for the IPS moral psychology, the attention to interpersonal and mutual ethical growth requires intimacy growth over time. The development of intimacy allows connecting a person’s own uniqueness as a human being with another’s. Empathy is a crucial element for this process as the core foundation of personal action in interpersonal relationships through personal free self-giving (freedom for) emphasizing personal growth through relational intimacy and not-autonomously as part of the very constitution of human beings in ontological-philosophical anthropology terms (Polo, 2015).

Fourthly, in IPS, action with relational intimacy aspires to a humanist transcendental-personalist perspective as understood in the philosophy of Polo (see Akrivou et al., 2022), whereby integrity seeks to enable both improving a focal IR while also striving for a sustainable wider co-existence. To this end, action requires finding an appropriate kind of personal practical wisdom which is more than just prudence, as it integrates the cognitive, affective, ethical, and practical bases and forms of knowing (Akrivou and Scalzo, 2020; Akrivou et al., 2020a,b). This practical wisdom is also avoiding the integrity of the autonomous self and its limitations (Akrivou and Orón, 2016).

*IPS considers empathy as part of personal action striving for integrity within a relational space which is characterized by free voluntary acts rooted in the person’s intimacy. Thus, intimacy guides voluntary action (freedom for) in a complicated two-way interaction that is managed by meta-level personal striving to reach out to specific others, and which is a continuity over context, time and each specific other.*

Based on the above analysis, IPS brings empathy as part of personal action striving for integrity within a relational space characterized by free voluntary acts rooted in the person’s intimacy which guides voluntary action (*freedom for*). This conceptualization implies a complicated two-way interaction which is guided by meta-level personal striving to reach out to specific others. Moreover, empathy in IPS is not one singular act, but a continuity over context, time and each specific other, and it is purposeful, as well.

In IPS, empathy is a sensitive process felt from within personal intimacy as part of the very integrity and self-integration of each human being; it aims toward co-existence and co-growth in the logic of personal gift love. In sum, empathy is crucial to how the self is understood in philosophical psychology and anthropology terms summarized in the previous paragraphs: it can support relational resilience and interpersonal growth without degradation of the “other’s personhood” but to enable both acceptance of the other and personal and shared integrity.

### 6.1. How IPS views empathy

For IPS, empathy is a complex two-way interaction that is guided by meta-level principles. Herein, empathy is not algorithmic because, when we deal with the other as a simple algorithm, we take the focus away from the person and our free self-giving, and place it on the mastery of the other and of ourselves (i.e., we tend toward the autonomous or processual-self path). IPS presents empathy as a sensitive process that emerges from within personal intimacy as part of each human being’s integrity and self-integration. Empathy is crucial to the understanding of the self in the terms of philosophical psychology and anthropology as outlined in the previous sections. Empathy supports the self-giving act, intentionally utilized to build bridges between the self and the other’s act of being. It can also support relational resilience and interpersonal growth without degrading the other’s personhood, and it can enable both acceptance of the other and personal and shared integrity. Therefore, IPS understands empathy as the effect of recognizing ourselves as internally linked by our constitutive relational reality. In some ways, we recognize ourselves in those with whom we are linked: “I recognize the other as my good because of our connection.” This is a pre-psychological reality in that it is not a creation of the person, but rather a recognition that occurs in the person. The psychological activity that we call empathy derives from such pre-psychological reality.

### 6.2. Empathy in IPS

Looking to the IPS theory and its premises, empathy in IPS is vital. According to IPS, the empathic act has at least seven distinctive aspects:

1. Empathy involves freely offering oneself and one’s work to the other person but also accepting the free response of the other as a person, and it is reinforced by personal integrity.

At an early stage of an IR, empathy involves freely offering oneself and one’s work to the other; it is reinforced by personal integrity and is not an imposed or mechanical act. Offering oneself and one’s work and accepting the free response of the other persons take part of the same relational reality. Although empathy is more required at an early stage to initiate personal relationships and the process of mutual growth, it is also important when accepting and receiving another person’s intimacy, inherent in the act of giving. Empathy acknowledges the free response of the other as person (i.e., without reducing or instrumentalizing the other), but it is assumed that this situation is far from perfect, as part of human imperfection and vulnerability. So empathetic response to the other as free system is not idealized but is never losing value of the other as person.

2. Empathy supports the IR constituting a space of psychological security, where personal growth and intimacy increases are based on mutual work.

Empathy is needed to complement a spiritual orientation toward “seeing” work as an appropriate avenue to the other person, so part of the relationship is expressed through shared work-related action. IPS theorization is premised on the sharing of personal intimacy, a precursor for integrity. Empathic engagement implies the need to access the other’s intimacy, and actions aimed to take the relationship beyond the early stages of shared intimacies *via* working with another person. Caring about another person and our shared growth implies employing empathy to proceed with the shared intention of increasing intimacy while maintaining psychological safety for everyone involved. Understanding work from a metaphysical perspective implies the assumption that others give something of themselves to us through the labor they freely offer us, in the sense that working with and for others reveals an intentional act of co-existence and freedom (for; self-giving). Empathy allows for psychologically safe intensifications of intimacy, irrespective of one’s objective valuation of the worthiness of another’s work, for example, in terms of quality or quantity, or according to externally set standards and expectations. This is because in IPS work is not a metric of personal quality, but rather a path toward knowing the other better and enabling mutual growth. Here, in addition, empathy allows us to turn our attention to the other, not just ourselves, to engage in common work, and to realize how this work exposes us to the other’s personhood. Work-based empathy allows for the reception of acts of wider kindness, and the appreciation of the others’ self-giving and the giver’s intention, which further cultivates a shared feeling that self-giving is received and valued. Empathy here enables acceptance (of the other), appreciation and reciprocation in all possible ways, and the continuation of a shared commitment. It is a more sensitive two-way process.

3. Empathy helps us face complex situations in which mutual expectancies differ. Here, empathy requires displaying trust in the face of adversity and uncertainty, and patiently utilizing it to overcome misunderstandings in order to work and regenerate.

Empathy helps us face situations in which the other person’s intentions, behavior, and interiority are not a straight line in a given, evolving relational context. Empathy serves to discover and build a deeper relationship and mutual understanding that often require trust in the face of adversity and uncertainty, and, at times, overcoming misunderstandings. People navigating modern life are busy with multiple demands, while also facing the things happening to their bodies and souls as they age and go through life stages. Often, their families, jobs, and wider social roles are confronted with novelty and require a response. Here, empathy is essential for the acceptance and maintenance of trust. Empathy is needed to forgive, offer patience, and ask the right questions, while insisting on common work and projects. But one needs empathy to see the truth behind the veil, something that depends on each person’s intimate personhood, personal history, dispositions and subjectivity, and on how one finds a path to reach out to the other.

4. Empathy enables practical wisdom through the exchange of knowledge within the IR.

Empathy informs and enables, *via* safe human connection, the ongoing development of shared practical wisdom in an internally-focused IR context, where practical wisdom, in turn, enables personal and interpersonal growth. Empathy is particularly relevant in the support of the person acting in a relationship to avoid cunning or clever forms of practical-ethical reason in action and in intention (Akrivou et al., 2020b). Systematic development of appropriate kinds of practical wisdom in IRs enables the ongoing mutual growth in IRs. Consequently, emotional self-awareness, genuine self-reflection, and interpersonal communication, aimed at the person and virtuously oriented, are required.

5. Empathy is needed to accept people in an IR as they are.

Empathy allows for better integration of the uniqueness and distinctiveness of another person as a single being in their own right, as well as mutual intentional growth in IRs. Empathy enables the acceptance of the person as absolute value and in who they are, that the other is not just a person who is there for oneself only, but someone who also has agency and a different path, a person who has to face a variety of challenges related to their virtuous growth, while also remaining invested in mutual growth in that IR.

6. Empathy catalyzes the pooling of shared affective commitment resources in the self, with an orientation toward another person, and supports shared intentionality when an IR or interpersonal growth is stagnant or negative.

Empathic acts help persons involved in both figuring out how to stimulate and improve the quality of their relationships and furthering mutual growth in their IRs. Therefore, empathy here helps one of the two interrelated parties to positively reawaken the other’s self-giving process or helps maintain their strong commitment to the further growth of their IR with and for the other. Empathic acts may not be visible actions, and can instead involve allowing room for solitude, breaks, and silence, or simply avoiding actions that may hurt the relationship or any party in it. Empathy is needed to become or remain balanced and wise is when it is tough to do so in relational and personal terms. We need empathy in response to “negative,” more distanced, or quieter loops in a relationship.

7. Empathy represents the hallmark of an IR, enabling shared solidarity and mutual support over time while accepting each person in their distinctiveness, as a complex and integrated being.

Empathy helps empathizers maintain a loving focus of action that has the power to remain resilient over time. This applies to the response from both parties in intimate IRs, free from the rejection or instrumentalization that might adversely impact their humanity and well-being. The empathic act is relied on to enable the development of each party’s shared interiority/intimacy and integrity, and the flourishing of shared solidarity and mutual support over time, which includes empathy informing prior and future personal action.

Table 1 evaluates current key empathy frameworks from the IPS perspective as well as summarizes the extent to which each empathy model captures and takes into account the seven key ways the IPS perspective conceptualizes empathy. In an attempt to represent an overall assessment, we associate with a binary outcome the seven distinctive potentialities of empathy with current theory. Based on this information, it can be established that although some of the IPS proposals are taken into account in the available models of empathy, some others (such as 4, 5, and 7) are not captured by any. This result highlights the complexity of empathy understood from the IPS perspective, which exceeds the actual unilateral visions used to theoretically and procedurally explain empathy nowadays, and justifies the need for an integrative view of empathy in the framework of IRs (Cikara and Van Bavel, 2014).

**Table 1:** Explanatory models of empathy from the perspective of IPS.

Empathy in IPS	Cyclical model of empathy Barrett-Lennard (1981)	Perception-action model Preston and De Waal (2002)	Multidimensional model Decety and Jackson (2004)	Self to other model Bird and Viding (2014)	Dual system model Heyes (2018)	Integrative hierarchical model Schurz et al. (2021)	Critical comment between models
1. Empathy involves freely offering oneself and one’s work to the other person, but also accepting the free response of the other as a person, and it is reinforced by personal integrity.	✅	✅	✅	✅	✅	❌	The models that include this first aspect believe that empathy is essential for the IR but do not emphasize its participation in the personal growth of the actors in the relationship.
2. Empathy supports the IR, constituting a space of psychological security where personal growth and intimacy increases are based on mutual work.	❌	❌	❌	✅	✅	❌	The models see the interrelated participation of the different components of empathy as able to account for the potential of human empathy, but these components explain empathic situations as concrete or discrete situations, and not as cumulative or continuous (in terms of intimacy) within an IR.
3. Empathy helps us face complex situations in which mutual expectancies differ. Here, empathy requires displaying trust in the face of adversity and uncertainty, and patiently utilizing it to overcome misunderstandings in order to work and regenerate.	✅	❌	✅	✅	✅	✅	Most models describe the components as interactive or cyclical processes. Since the Perception-Action Model involves some chaos and its explanation of empathy is an adaptation of motor processes, we believe its nature is more mechanistic than linear.
4. Empathy enables practical wisdom through the exchange of knowledge within the IR.	❌	❌	❌	❌	❌	❌	The models do not examine, nor are they concerned about, the role of empathy in avoiding vice and seeking virtue by each party in an IR.
5. Empathy is needed to accept people in an IR as they are.	❌	❌	❌	❌	❌	❌	The models do not examine nor are they concerned about concrete IR contexts, the distinctiveness and uniqueness of each person, or with the distinct blueprint that each participant shares in a relational context.
6. Empathy catalyzes the pooling of shared affective commitment resources in the self, with an orientation toward another person, and supports shared intentionality when an IR or interpersonal growth is stagnant or negative.	❌/✅	❌/✅	❌/✅	❌/✅	❌/✅	❌/✅	Although the different models do not specifically include relational aspects in the context of an empathic act, they could be considered a simplified explanation of the empathic acts emitted by the parties within an adverse context such as a negative IR. However, this does not explain the complexity of the interaction between the empathic act and the context in which it occurs.
7. Empathy represents the hallmark of an IR, enabling shared solidarity and mutual support over time while accepting each person in their distinctiveness, as a complex and integrated being.	❌	❌	❌	❌	❌	❌	None of the models specifically include relational aspects in the context of an empathic act or pay attention to how to maintain a loving focus relevant to the distinctiveness of the other as they change over time in terms of how they respond in intimate IRs.
Critical comment regarding each of the models (summary review of each model from the IPS perspective)	This model delineates a sequence of distinct stages in empathic interaction: empathic resonation, responsive understanding, and actual reception or awareness of communication.	This model, together with an understanding of how representations change with experience, can explain the effects of similarity, familiarity, past experience, explicit teaching, and salience.	This model emphasizes the dynamic interaction of cognitive and affective states *via* cognitive mastery, thus making the empathic act possible.	This model gives importance to the information- processing steps necessary for the instantiation of empathy.	This model suggests that empathy depends on a matching mechanism constructed as associative learning develops.	This model is composed of empathy-based task components that do not provide relevant information on key aspects related to interpersonal growth in IPS.	

## 7. Conclusion

Research on empathy in psychology has been on the rise in recent decades. Various theories of empathy and related operationalization efforts have significantly informed psychology’s theoretical and practical research, both regarding its role and significance. Yet, we argue that there is still room for further research to help fully capture the important notion of empathy and its theoretical and conceptual density. This contribution offers a critical overview of current research that conceptualizes and measures empathy and suggests the importance of a shared vision of empathy, as well as its relevance for psychology and neuroscience. We also argue for the relevance of shared intentionality and vision in empathy-related actions and, upon review of other models, with emphasis on a shared vision that informs research on empathy. In this respect, we suggest that a newly-developed theory of self, human growth, and action, the so-called Inter-Processual Self, or IPS, can novelly bolster empathy theory in order to go beyond what has been proposed in the literature.

After offering a systematic analysis of IPS, and its understanding of empathy, with a focus on the interpersonal relational growth of all parties involved, we have presented seven distinctive perspectives that this theory offers to empathy research. Furthermore, we have examined the most prominent contemporary theories on empathy and concluded that they only partially cover IPS’s view of empathy in terms of the self, action, and human growth. These aspects of empathy are key because they capture their essential role in interpersonal relationships. Therefore, theories on empathy should be informed by the novel perspective presented here, which is based on how IPS understands integrity as a relational act, where empathy is an essential mechanism. Specifically this distinctive proposal can orient future theory toward a more novel direction to better capture (a) its participation in personal growth; (b) the link between the interrelational components of empathy to explain empathic situations in a cumulative or continuous manner within an interpersonal relationship; (c) its nature, which transcends a mechanistic or linear understanding of the term; (d) its role in character development in interpersonal relations and, particularly, in helping avoid vice and seek virtue *via* interpersonal and relational integrity-based actions; (e) its role in concrete distinctive contexts, understanding the distinctiveness and uniqueness of each person in a relational context; (f) its role and relevance in an adverse context, such as a negative interpersonal relationship and complex situations; and (g) its role in maintaining a loving focus of action that has the power to maintain resilience and a qualitative approach over time, thus helping all parties in intimate interpersonal relations respond without rejection or instrumentalization, which would have adverse effects on their humanity and well-being.

The present contribution has been made in order to enrich the current theories on empathy, as well as to inform future research directions. Despite related advances, future models are still faced with the challenge of capturing information about the interpersonal processes that take place when empathy is present. Considering that individuals’ responses to contexts can differ and thus have distinct implications for interlocutors, it is important that paradigms or approaches based on empathy are able to integrate participants’ responses in different contexts.

## Data availability statement

The original contributions presented in the study are included in the article/Supplementary material, further inquiries can be directed to the corresponding author.

## Author contributions

EL, MM, and KA: conceptualization, formal analysis, methodology, validation, writing—original draft, and writing—review and editing. GS, MA, and JO: writing—original draft and writing—review and editing. All authors contributed to the article and approved the submitted version.

## Conflict of interest

The authors declare that the research was conducted in the absence of any commercial or financial relationships that could be construed as a potential conflict of interest.

## Publisher’s note

All claims expressed in this article are solely those of the authors and do not necessarily represent those of their affiliated organizations, or those of the publisher, the editors and the reviewers. Any product that may be evaluated in this article, or claim that may be made by its manufacturer, is not guaranteed or endorsed by the publisher.
