# Supplementary material for: The role of empathy in shared intentionality: Contributions from Inter-Processual Self theory
Source: Front Psychol. 2023 Mar 10;14:1079950. doi: 10.3389/fpsyg.2023.1079950 (PMC10036387; doi:10.3389/fpsyg.2023.1079950)
Supplement: Supplementary file 1 [file Data_Sheet_1.pdf]

Supplementary Material

1. Tables

Supplementary Table 1. Characteristics of self-report questionnaires (SRQs) for assessing empathy in the general population.

**Instrument**	**Description**	**Scale components**	**Scale properties**
Hogan's Empathy Scale (Hogan, 1969)	64-item SRQ that evaluates empathy, defined as "*the intellectual or imaginative apprehension of another's condition or state of mind*".	Social self- confidence, even temperedness, sensitivity, and nonconformity.	Dichotomous
Emotional Empathic Tendency Scale (Mehrabian & Epstein, 1972)	33-item SRQ designed to assess emotional empathy, defined as "*a vicarious emotional response to the perceived emotional experiences of others*."	Susceptibility to emotional contagion, appreciation of the feelings of unfamiliar and distant others, extreme emotional responsiveness, tendency to be moved by others' positive emotional experiences, tendency to be moved by others' negative emotional experiences, sympathetic tendency, willingness to be in contact with others who have problems.	9-point Likert rating
Interpersonal Reactivity Index (Davis, 1983)	28-item SRQ that assesses several psychological aspects of cognitive and emotional empathy, while understanding empathy as a complex multidimensional concept.	Empathic concern, perspective taking, personal distress, and fantasy.	5-point Likert rating
Balanced Emotional Empathy Scale (Mehrabian, 1996)	30-item SRQ that measures emotional empathy, defined as an '*increased responsiveness to another's emotional experience*'.	Unidimensional measure of emotional experience.	9-point Likert rating
Multidimensional Emotional Scale (Caruso & Mayer, 1998)	30-item SRQ measure of empathy which yields scores measuring multiple dimensions of emotional empathy.	Suffering, positive sharing, responsive crying, emotional attention, feeling for others, emotional contagion.	5-point Likert rating
Empathy Quotient (Baron-Cohen & Wheelwright, 2004)	60-item SRQ to measure empathy, understood as '*the drive to identify another person's emotions and thoughts, and to respond to these with an appropriate emotion*'.	Cognitive empathy, emotional reactivity, social skills.	4-point Likert rating
Basic Empathy Scale (Jolliffe & Farrington, 2006)	40-item SRQ measure of empathy, conceptualized as '*sharing and understanding of another's emotional state or context resulting from experiencing the emotive state and understanding another's emotions*'.	Cognitive empathy, affective empathy.	5-point Likert rating
Toronto Empathy Questionnaire (Spreng et al., 2009)	16-item SRQ representing empathy as a primarily emotional process.	Unidimensional measure of empathy.	5-point Likert rating
Empathy Assessment Index (Lietz et al., 2011)	17-item SRQ based on a comprehensive definition of empathy, conceptualized as the "ability to understand the circumstances of other people's living conditions in the context of broader educational, health, and socioeconomic structures and institutions".	Affective response, self-other awareness, emotion regulation, perspective taking, empathic attitudes.	6-point Likert rating

Questionnaire of Cognitive and Affective Empathy *(Reniers et al., 2011)*	31-item SRQ specific to cognitive and affective components of empathy.	Perspective taking, online simulation, emotion contagion, proximal responsivity, peripheral responsivity,	4-point Likert rating
Light-Moran Positive Empathy Scale (Light et al., 2019)	15-item self-report questionnaire measuring positive-valence empathy.	Emphatic happiness, emphatic cheerfulness.	7-point Likert rating

The role of empathy in shared intentionality

Supplementary Table 2. Description of behavioral approaches for assessing empathy (ordered by publication date).

**Instrument**	**Objective**	**Variables**
Empathy in Children (Feshbach & Roe, 1968)	To investigate stimulus conditions influencing empathic responses in first-grade children, where empathy is defined as a vicarious response.	Children's comprehension of affective situations, the presence of the appropriate labels in their verbal repertoire.
Barrett-Lennard Relationship Inventory (Barrett-Lennard, 1962)	To examine the client's perceptions of the therapist's attitudes in the relationship, based on client and therapist ratings.	Empathic understanding, level of regard, unconditionally of regard, congruence.
Accurate Empathy Scales (Truax & Carkhuff, 1964)	To operationalize empathy into specific behaviors and statements.	The nature of the scale is indicated by giving the definition of Stage 1 (i.e., lowest level of empathic understanding) to Stage 9 (i.e., very high degree of empathy).
Response Empathy Rating Scale (Elliot et al., 1982)	To measure the empathic quality of counselor behavior, based on observer-rating.	Depth expressiveness and empathic exploration.
Dyadic interaction paradigm (Ickes et al., 1988)	To study the intersubjective phenomenon of empathic accuracy, which refers to the therapist-client perceptual congruence.	Valence accuracy, and content accuracy.
Concerns for others scale (Zahn-Waxler et al., 1992)	To explore the affective, behavioral, and cognitive components of children's responses to others' distress.	Prosocial behavior, empathic concern, hypothesis testing, self-referential behaviors, self-distress, aggressive behavior, and positive affect.
Standard Stimulus Paradigm (Marangoni et al., 1995)	To infer the specific content of the thoughts and feelings of the same set of target persons whom they view in a standard set of videotaped interactions.	Extent to which individual perceivers accurately infer the thoughts and feelings of the same target.
Faux pas task (Stone et al., 1998)	To test subjects' ability to infer that someone can have a mistaken belief that is different from their own true belief.	Number of correct answers.
Emotional attribution task (Blair & Cipolotti, 2000)	To investigate the ability to attribute emotional states to others.	Number of correct attributions.
Measure of expressed empathy (Watson & Prosser, 2002)	To measure therapists' verbal and non-verbal behaviours, speech characteristics, and response modes. Based on observer-rating.	Therapist's concern for the client, expressivity of voice, capturing the intensity of client feelings, warmth, attunement to the client's inner world, communicating an understanding of the client's meanings or cognitive framework, communicating understanding of the client's feelings and inner experiences, responsiveness to the client, and looking concerned in facial expression or body posture.
Consultation And Relational Empathy (Mercer et al., 2004)	To develop a consultation process measure based on a broad definition of empathy, in the context of the clinical encounter.	Patient perception of relational empathy in the consultation.
Comic strip task (Völlm et al., 2006)	To make a decision in non-verbal task that presents a series of comic strips and asks participants to choose the best one out of two or three strips on an answer card to finish the story.	Accuracy in assessing other individuals' mental states.
Picture viewing paradigms (Westbury & Neumann, 2008)	To examine how indices related to human empathic responding vary across empathy-eliciting scenarios depicting victimized circumstances that were distressing, violent, or oppressive.	Empathy is conceptualized as an individual's self-reported response to empathy-eliciting visual images.

Picture Story Stimuli<br>*(Nummenmaa et al., 2008)*	To test whether emotions triggered by affect-laden events in movies are associated with synchronization of viewers' brain activity.	Empathy is conceptualized as the ability to interpret visual scenes and predict the most likely behavioral consequence based on cognitive or affective cues.
Kids' Empathetic Development Scale<br>*(Reid et al., 2012)*	To assess cognitive, affective, and behavioral components of empathy in kids.	Cognitive, affective, and behavioral components of empathy are examined using emotion recognition, picture-based scenarios, and behavioral self-report techniques.
Therapist empathy scale<br>*(Decker et al., 2014)*	To assess affective, cognitive, attitudinal, and attunement aspects of therapist empathy.	Concern, expressiveness, resonate, warmth, attuned to client's inner work, and understanding cognitive framework.

References

Baron-Cohen, S. and Wheelwright, S. (2004). The empathy quotient: an investigation of adults with Asperger syndrome or high functioning autism, and normal sex differences. Journal of autism and developmental disorders, 34(2), 163-175.

Barrett-Lennard, G.T. (1962). Dimensions of therapist response as causal factors in therapeutic change. Psychological monographs: General and applied, 76(43), 1.

Blair, R.J. and Cipolotti, L. (2000). Impaired social response reversal: A case of acquired sociopathy'. Brain, 123(6), 1122-1141.

Caruso, D. R., and Mayer, J. D. (1998). A measure of emotional empathy for adolescents and adults.

Davis, M.H. (1983). Measuring individual differences in empathy: Evidence for a multidimensional approach. Journal of personality and social psychology, 44(1), 113.

Decker, S.E., Nich, C., Carroll, K.M. and Martino, S. (2014). Development of the therapist empathy scale. Behavioural and cognitive psychotherapy, 42(3), 339-354.

Elliott, R., Filipovich, H., Harrigan, L., Gaynor, J., Reimschuessel, C., and Zapadka, J. K. (1982). Measuring response empathy: The development of a multicomponent rating scale. Journal of counseling psychology, 29(4), 379.

Feshbach, N. D., and Roe, K. (1968). Empathy in six-and seven-year-olds. *Child development*, 133-145.

Hogan, R. (1969). Development of an empathy scale. Journal of consulting and clinical psychology, 33(3), 307.

Ickes, W., Tooke, W., Stinson, L., Baker, V. L., and Bissonnette, V. (1988). Naturalistic social cognition: Intersubjectivity in same-sex dyads. Journal of Nonverbal Behavior, 12(1), 58-82.

Jolliffe, D. and Farrington, D.P. (2006). Examining the relationship between low empathy and bullying. Aggressive Behavior: Official Journal of the International Society for Research on Aggression, 32(6), 540-550.

Lietz, C. A., Gerdes, K. E., Sun, F., Geiger, J. M., Wagaman, M. A., and Segal, E. A. (2011). The Empathy Assessment Index (EAI): A confirmatory factor analysis of a multidimensional model of empathy. Journal of the Society for Social Work and Research, 2(2), 104-124.

Light, S. N., Moran, Z. D., Zahn-Waxler, C., and Davidson, R. J. (2019). The measurement of positive valence forms of empathy and their relation to anhedonia and other depressive symptomatology. Frontiers in Psychology, 10, 815.

Marangoni, C., Garcia, S., Ickes, W., and Teng, G. (1995). Empathic accuracy in a clinically relevant setting. Journal of personality and social psychology, 68(5), 854.

The role of empathy in shared intentionality

Mehrabian, A., and Epstein, N. (1972). A measure of emotional empathy. Journal of personality.

Mehrabian, A. (1996). Manual for the balanced emotional empathy scale (BEES). Available from Albert Mehrabian, 1130.

Mercer, S. W., Maxwell, M., Heaney, D., and Watt, G. (2004). The consultation and relational empathy (CARE) measure: development and preliminary validation and reliability of an empathy-based consultation process measure. Family practice, 21(6), 699-705.

Nummenmaa, L., Hirvonen, J., Parkkola, R. and Hietanen, J.K. (2008). Is emotional contagion special? An fMRI study on neural systems for affective and cognitive empathy. Neuroimage, 43(3), 571-580.

Reid, C., Davis, H., Horlin, C., Anderson, M., Baughman, N., and Campbell, C. (2013). The Kids' Empathic Development Scale (KEDS): A multi-dimensional measure of empathy in primary school-aged children. British Journal of Developmental Psychology, 31(2), 231-256.

Reniers, R. L., Corcoran, R., Drake, R., Shryane, N. M., and Völlm, B. A. (2011). The QCAE: A questionnaire of cognitive and affective empathy. Journal of personality assessment, 93(1), 84-95.

Spreng, R. N., McKinnon, M. C., Mar, R. A., and Levine, B. (2009). The Toronto Empathy Questionnaire: Scale development and initial validation of a factor-analytic solution to multiple empathy measures. Journal of personality assessment, 91(1), 62-71.

Stone, V.E., Baron-Cohen, S. and Knight, R.T. (1998). Frontal lobe contributions to theory of mind. Journal of cognitive neuroscience, 10(5), 640-656.

Truax, C. B., Carkhuff, R. R., and Douds, J. (1964). Toward an integration of the didactic and experiential approaches to training in counseling and psychotherapy. Journal of Counseling Psychology, 11(3), 240–247.

Völlm, B.A., Taylor, A.N., Richardson, P., Corcoran, R., Stirling, J., McKie, S., Deakin, J.F. and Elliott, R. (2006). Neuronal correlates of theory of mind and empathy: a functional magnetic resonance imaging study in a nonverbal task. Neuroimage, 29(1), 90-98.

Watson, J. C., and Prosser, M. (2002). Development of an observer rated measure of therapist empathy. Client-centered and experiential psychotherapy in the 21st century: Advances in theory, research and practice, 303-314.

Westbury, H. R., and Neumann, D. L. (2008). Empathy-related responses to moving film stimuli depicting human and non-human animal targets in negative circumstances. Biological psychology, 78(1), 66-74.

Zahn-Waxler, C., Radke-Yarrow, M., Wagner, E. and Chapman, M. (1992). Development of concern for others. Developmental psychology, 28(1), 126.
